# Comparative structural and functional analysis of the PGU1 protein from *Saccharomyces bayanus* with other *Saccharomyces species*

**DOI:** 10.6026/97320630018464

**Published:** 2022-05-31

**Authors:** Chennupati Vidyasagar, Pasupuleti Santhosh Kumar, Poondla Vijayakumar, Saggam Alekya, Katike Umamahesh, Obulam Vijaya Sarathi Reddy

**Affiliations:** 1 Department of Biochemistry, S.V. University, Tirupati 517 502, India; 2 Department of Biotechnology, SVIMS University, Tirupati 517 502, India

**Keywords:** Saccharomyces bayanus, PGU1, Pectin, Homology modelling, Molecular docking

## Abstract

An endo-poly-galacturonase (PGU1) gene product is responsible for the pectolytic activity in Saccharomyces bayanus. Therefore, it is of interest to document the comparative structural and functional analysis of the PGU1 protein from Saccharomyces bayanus with those in other Saccharomyces related species. The molecular docking analyses of pectin with the different homology models of PGU1 protein from several Saccharomyces species are reported.

## Background:

Polygalacturonases (PGs) are enzymes that degrade pectin [1]. PGs are involved in breaking of α-1, 4-glycosidic bonds between the residues of two galacturonic acids [2]. PGs are classified by their activity as endo-polygalacturonases and exo-polygalacturonases and these are involved in the hydrolysis of polygalacturonic acid and release oligosaccharidic chains in various lengths and after canalization, they release single galacturonic acid residues [3]. Production and characterisation of PGs have been reported in many plants, fungi, yeast and bacterial species [4-5]. Microorganisms are sources of many enzymes [5,6]. Among yeast Saccharomyces cerevisiae is also produce PG's [7]. Polygalacturonase (PGU) gene is a part of the genome of many bacterial, fungal and plant species. Phenotypes of these genes are to express pectinase enzyme [8]. The PGU1, PGL1 and sp. PGU genes consist of a 1086 bp ORF, encoding a 361 AA polypeptide chain, with the molecular mass of 37,288 Da and theoretical PI of 8.55, these genes show few single nucleotide sequence polymorphism [8-10]. The first 18 amino acids act as signal peptide sequences and consist of Ala residue as the cleavage site [11]. The X-ray crystallography was used to study the substrates binding sites of enzymes [12]. The application of molecular modelling in the understanding of protein function from sequence is well known [13]. Therefore, it is of interest to document the comparative structural and functional analysis of the PGU1 protein from Saccharomyces bayanus with those in other (selective) Saccharomyces species. The molecular docking analyses of pectin with the different homology models of PGU1 protein from several Saccharomyces species are also reported.

##  Materials and Methods:

### Sequence retrieval alignment:

The Saccharomyces bayanus PGU1 nucleotide sequence was obtained from NCBI data base (GenBank: FR847039.1) and The complete amino acid sequence of this protein (F8KAD0) was retrieved from uniport sequence database in FASTA format [13] (http://www.uniprot.org/) and it was used for homology structure building.

### Homology modelling of Saccharomyces bayanus PGU1:

The NCBI Basic Local Alignment Search Tool (BLAST) for the sequence similarities used for searching the crystal structure of the closest homologs available in the Brookhaven Protein Data Bank (PDB). Based on maximum identity with high score and lower e-value, endo-polygalacturonase from Colletotrichum lupini (PDB code: 2IQ7) with a resolution of 1.9A used as template, and 3D homology model was built by using SWISS-Model [14,15].

### Validation of 3D structure:

The quality of the structure was validated using PROCHECK and ProSAweb servers. Ramachandran plot used to access the quality of the model by looking into the allowed and disallowed regions of the plot. Z-score values were generated from the ProSAweb server which determines the overall quality of the model and identity nearest to the native NMR/X-ray crystal structure [15].

### Structural comparison of S. bayanus PGU1 with other Saccharomyces PGU1:

The comparative structural analysis studies were used to ensure the identity and variability of S. bayanus PGU1 structure with other Saccharomyces PGU1 structures using the PyMol software program which define the structural similarity score as the log-odds of two probabilities using a scheme similar to Dayhoff’s amino acid substitution score. An alignment of superimposed structures and similarities were predicted as scores and RMSD values for the following structures: the validated 3D model of S. bayanus PGU1 was superimposed with other Saccharomyces PGU1 structures such as Saccharomyces cerevisiae PGU1 (built structure), Saccharomyces paradoxus PGU1 (built structure), Saccharomyces pastorianus PGU1 (built structure), Saccharomyces uvarum pgu1 CLIB 113 (built structure) and Saccharomyces uvarum PGU1 CBS 395T (built structure), were recorded [16]. For those PDB structures that were not available, the 3D structures were built by using the method mentioned earlier (Data was not shown).

### Molecular docking:

It was carried out using the MOE docking software tool (MOE 2011.10). The 3D structure of pectin retrieved from PubChem [17] and its geometry was optimized in a MOE working environment. The S. bayanus PGU1 and other Saccharomyces PGU1 structures were loaded individually into MOE software removing water molecules and heteroatoms, polar hydrogens were added. The structures were protonated at a temperature of 300K, pH 7 and a salt concentration of 0.1. The generalized born implicit solvating environment was enabled with a dielectric constant of 1 and Van der Waals forces were enabled at a cut off value of 10 Å. Energy minimization carried out in the OPLS force field at a gradient of 0.05 to calculate the atomic coordinates of the protein that are local minima of the molecular energy function and to determine low energy conformations; molecular dynamics simulations were carried out in the same force field. NVT statistical ensemble was used for the temperature held fixed to generate the trajectories. The most accurate Nose-Poincare-Anderson algorithm was enabled to solve the equation of motion during simulations. The initial temperature was set at 30 K and increased to a run time temperature of 300 K and the simulations were carried out for a total period of 10 ns and the stabilized conformations generated at the end of the simulations were used for the molecular docking process. Individual dockings were performed for S. bayanus PGU1 and other Saccharomyces PGU1 with pectin to find out the binding modes and affinity variations. These docked conformers were generated by superposition of ligand atom triplets and triplets of receptor site points using alpha triangle docking placement methodology. The docked conformers were ranked by the London dG scoring function to estimate the free energy of binding of the ligand from a given pose. The conformations thus were refined and re-scored in the same force filed to remove the duplicate conformations. At the end of the docking process, the pose with the least score was chosen from the total conformations and in each docking process, the binding orientations of glucose were studied in the binding sites of S. bayanus PGU1 and other Saccharomyces PGU1 [18,19].

## Results and Discussion:

The crystal structure of S. bayanus PGU1 generated by using Swiss Model. The X‑ray crystallographic structure of endo polygalacturonase from the phytopathogenic fungus Colletotrichum lupini (PDB ID: 2iq7) was used as template which showed 57.2% sequence identity (Figure 1 - see PDF). The build S. bayanus PGU1 of the final model (Figure 2 - see PDF) was verified by submitting the build S. bayanus PGU1. pdb file to PROCHECK validation server and Ramachandran plot showed 85.4% of the residues were found in the most favourable allowed regions [20]. Moreover, the ProSA‑Web evaluation of build S. bayanus PGU1 model revealed a compatible Z-score value that falls in the range of native conformations of X‑ray crystal structure [21,22]. The BLAST results of the S. bayanus PGU1 gene sequence revealed close homology with other Saccharomyces PGU1 sequences indicates that the sequence is highly conserved among all the species (Figure 3 - see PDF). Further, the PGU1 sequence showed a distinct poly-galacturonase active site at the region (215 – 228) amino acids, which is indicated as three conserved residues in the motif NNYCYNGHGISIGS among all species. The multiple sequence alignment of S. bayanus PGU1 results indicated differences with other Saccharomyces PGU1 sequences and till now there is no crystal structure of S. bayanus PGU1 in the PDB database [16]. The S. bayanus PGU1 structure showed 100% homology with Saccharomyces uvarum PGU1 and Saccharomyces pastorianus PGU1 with the RMSD values being 0.00 Å and 0.00 Å respectively. A Saccharomyces cerevisiae Pgu1 with the RMSD values RMSD 0.021, Saccharomyces paradoxus PGU1 with the RMSD values 0.028 and Saccharomyces uvarum CLIB113 PGU1 structures showed extensive variations both in the domain and non-domain regions values (Figure 4 - see PDF). The identical regions were random throughout the alignment especially in the structural superimposition of substrate binding regions showed much extent of variation and completely showing different conformations as indicated from the RMSD [17]. The docking of pectin into the substrate-binding sites of S. bayanus PGU1 and S. bayanus PGU1 structures revealed variable binding orientations along with different docking scores were depicted in Table 1(see PDF). The lowest docking score indicates the higher stability of the ligand enzyme complex [17]. The docking scores indicate that S. bayanus PGU1 forms a more stable complex with pectin compared with other Saccharomyces PGU1 structures. It represents that particular polygalacturanases having pectin degrading activity [23]. Pectin is found to be interacting with ASP182, ASN206, ARG282 and SER228 in S. bayanus PGU1 forming a total of 8 hydrogen bonds (Figure 5 - see PDF). These results conclude that the higher affinity of pectin towards PGU1 compared with other Saccharomyces PGU1 structures, strongly suggest that prominent pectolytic activity was observed in S. bayanus.

## Conclusion:

We document the comparative structural and functional analysis of the PGU1 protein from Saccharomyces bayanus with those in other Saccharomyces species. The molecular docking analyses of pectin with the different homology models of PGU1 protein from several Saccharomyces species are shown good binding affinity. This work is helpful to explore in gene cloning and expression virtually.

## References

[1] Hoondal GS (2002). Appl Microbiol Biot.

[2] Fogarty WM, Kelly CT (1983). In Microbial Enzymes and Biotechnology.

[3] Maness NO, Mort AJ (1989). Anal Biochem.

[4] Stratilová E (1993). J protein chem.

[5] Obafemi YD (2019). Int J Microbiol.

[6] Jayani RS (2005). Process Biochem.

[7] Blanco P (1997). Arch Microbiol.

[8] Naumov GI (2016). Microbiol.

[9] Blanco P (1998). FEMS Microbiol Lett.

[10] Gognies S (1999). Yeast.

[11] Hou W-C (1999). Bot Bull Academic Sin.

[12] Sasin JM (2004). Nucleic Acids Res.

[13] Bandikari R (2016). Current Trends in Biotech Pharmacy.

[14] Berman HM (2000). Nucleic Acids Res.

[15] Waterhouse A (2018). Nucleic Acids Res.

[16] Lakshmi HP (2013). Bioinformation.

[17] Kumar PS (2014). Indian J Pharm Sci.

[18] Kawabata T. (2003). Nucleic Acids Res.

[19] Wang Y (2009). Nucleic Acids Res.

[20] Kamata K (2004). Structure.

[21] NandaKumar Y (2012). Bioinformation.

[22] Laskowski RA (1996). J Biomol NMR.

[23] Poondla V (2017). 3 Biotech.

